# Two-step nationwide epidemiological survey of myasthenia gravis in Japan 2018

**DOI:** 10.1371/journal.pone.0274161

**Published:** 2022-09-21

**Authors:** Hiroaki Yoshikawa, Yumi Adachi, Yosikazu Nakamura, Nagato Kuriyama, Hiroyuki Murai, Yoshiko Nomura, Yasunari Sakai, Kazuo Iwasa, Yutaka Furukawa, Satoshi Kuwabara, Makoto Matsui, Taskforce of Validation of Evidence-based Diagnosis and Guidelines, and Impact on Quality of Life (QOL) in Patients with Neuroimmunological Diseases

**Affiliations:** 1 Health Service Center, Kanazawa University, Kanazawa, Ishikawa, Japan; 2 Jichi Medical University, Shimotsuke, Tochigi, Japan; 3 Department of Epidemiology for Community Health and Medicine, Kyoto Prefectural University of Medicine, Kamigyo-Ku, Kyoto, Japan; 4 Department of Neurology, School of Medicine, International University of Health and Welfare, Narita, Chiba, Japan; 5 Yoshiko Nomura Neurological Clinic for Children, Bunkyo-Ku, Tokyo, Japan; 6 Department of Pediatrics, Graduate School of Medical Sciences, Kyushu University, Fukuoka, Fukuoka, Japan; 7 Department of Neurology and Neurobiology of Aging, Kanazawa University Graduate School of Medical Science, Kanazawa, Japan; 8 Department of Neurology, Graduate School of Medicine, Chiba University, Chiba, Chiba, Japan; 9 Department of Neurology, Kanazawa Medical University, Uchinada, Ishikawa, Japan; PLOS ONE, UNITED KINGDOM

## Abstract

**Objective:**

To study the updated prevalence and clinical features of myasthenia gravis (MG) in Japan during 2017.

**Methods:**

We sent survey sheets to the randomly selected medical departments (number = 7,545). First, we asked the number of MG patients who visited medical departments from January 1, 2017, to December 31, 2017. Then, we sent the second survey sheet to the medical departments that answered the first survey to obtain the clinical information of patients who received MG diagnosis between January 1, 2015, and December 31, 2017.

**Results:**

The received answer to the first survey were 2,708 (recovery rate: 35.9%). After all, the prevalence of the 100,000 population was estimated as 23.1 (95%CI: 20.5–25.6). As a result of the second survey, we obtained 1,464 case records. After checking the duplications and lacking data, we utilized 1,195 data for further analysis. The median [interquartile range (IQR)] from the onset age of total patients was 59 (43–70) years old. The male-female ratio was 1: 1.15. The onset age [median (IQR)] for female patients was 58 (40–72) years old, and that for male patients was 60 (49–69) years old (Wilcoxon-Mann-Whitney test, p = 0.0299). We divided patients into four categories: 1) anti-acetylcholine receptor antibody (AChRAb) (+) thymoma (Tm) (-), 2) AChRAb(+)Tm(+), 3) anti-muscle-specific kinase antibody (MuSKAb) (+), and AChRAb(-)MuSKAb(-) (double negative; DN). The onset age [median (IQR)] of AChRAb(+)Tm(-) was 64 (48–73) years old, and AChRb(+)Tm(+) was 55 (45–66), MuSKAb(+) was 49 (36–64), DN was 47 (35–60) year old. The multivariate logistic regression analysis using sex, initial symptoms, repetitive nerve stimulation test (RNST), and edrophonium test revealed that sex, ocular symptoms, bulbar symptoms, and RNST were factors to distinguish each category. The myasthenia gravis activities of daily living profile at the severest state were significantly higher in MuSKAb(+). MuSKAb(+) frequently received prednisolone, tacrolimus plasmapheresis, and intravenous immunoglobulin; however, they received less acetylcholine esterase inhibitor. 99.2% of AChRAb(+)Tm(+) and 15.4% of AChRAb(+)Tm(-) received thymectomy. MuSKAb(+) did not receive thymectomy, and only 5.7% of DN received thymectomy. The prognosis was favorable in all categories.

**Conclusion:**

Our result revealed that the prevalence of Japanese MG doubled from the previous study using the same survey method in 2006. We also found that the onset age shifted to the elderly, and the male-female ratio reached almost even. Classification in four categories; AChRAb(+)Tm(-), AChRAb(+)Tm(+), MuSKAb(+), and DN, well describe the specific clinical features of each category and differences in therapeutic approaches.

## Introduction

Myasthenia gravis (MG) is an autoimmune disease that targets post-synaptic molecules at the neuromuscular junction [1, 2]. Historically, Patrick and Lindstrom reported that rabbits developed weakness after repeated immunization with acetylcholine receptor (AChR) protein purified from electric eels [3]. Lindstrom et al. reported the diagnostic value of anti-AChR antibody (AChRAb) in MG in 1976 [4]. Following the AChRAb, Hoch et al. found antibodies against muscle-specific kinase (MuSK) in 70% of AChRAb-negative MG patients [5]. The other candidates for autoimmune targets are low-density lipoprotein receptor-related protein 4 (LRP4) [6] and agrin [7]. However, the pathophysiological mechanism of these autoantibodies is not fully understood. Although the predominant IgG subclasses of AChRAb are 1 and 3 [8], anti-MuSK antibodies (MuSKAb) are subclass 4 [9]. The different IgG subclasses of autoantibodies suggest that the pathogenesis is different in the MG of AChRAb (+) and that of MuSKAb (+). Additionally, the accompanying thymic abnormalities (thymoma or thymic hyperplasia) are frequent in MG patients with AChRAb [10]. Interestingly, patients rarely have both AChRAb and MuSKAb [11]. On the other hand, AChRAb(-) MuSKAb(-) (double negative, DN) is another category [12]. The accumulating knowledge of autoantibodies requires us to understand MG by the autoantibody profiles. Moreover, thymoma is a unique feature for a subgroup of MG. Therefore, the analysis of patients compared with or without thymoma is meaningful because thymoma might have essential pathogenic roles in MG [13].

One of the methods to study the disease entity is an epidemiological study. It also contributes to establishing health care policies and helps clarify the etiology. Therefore, a periodic epidemiological survey is necessary to understand the alterations in the prevalence and features of diseases. Interestingly, an increasing incidence rate of MG was reported in Canada in the population over 65 years old without alteration of incidence in less than 64 years old between 1986 and 2006 [14]. Cortés-Vicente, E. et al. analyzed the data from the Spanish Registry of Neuromuscular Diseases classified into three age subgroups: early-onset MG (age at onset <50 years), late-onset MG (onset ≥50 and <65 years), and very-late-onset MG (onset ≥65 years) [15]. Eventually, they found that patients with MG were primarily ≥65 years of age with AChRAb and no thymoma.

There are two distinctive differences in MG’s medical practice in Japan from other countries. Firstly, government-assisted specialized neurologists and researchers guided the research and medical treatment in autoimmune neurological diseases. Ministry of Health, Labour, and Welfare of Japan (MHLW) designated MG as an intractable disease in 1972 that required continuous research and public support for patients’ welfare. The second feature of the medical practice was an early introduction of tacrolimus as a first-line therapy combined with prednisolone (PSL) [16]. After that, the emerging role of tacrolimus in MG became recognized [17].

Murai et al. performed the previous national survey of Japanese MG in 2006 [18]. Twelve years have passed since the last survey. The test of MuSKAb has been available in Japan since 2013. The availability of autoantibodies testing (AChRAb and MuSKAb) enables us to study the clinical characteristics of patients in detail. Besides, tacrolimus treatment has been covered by medical insurance since 2008. Therefore, it is meaningful to conduct a nationwide epidemiological study based on the same method in 2006. Our survey adds new findings for changing pictures of MG in Japan, which give insights to clinicians and researchers in other countries.

## Materials and methods

### Study design

**Fig 1:**
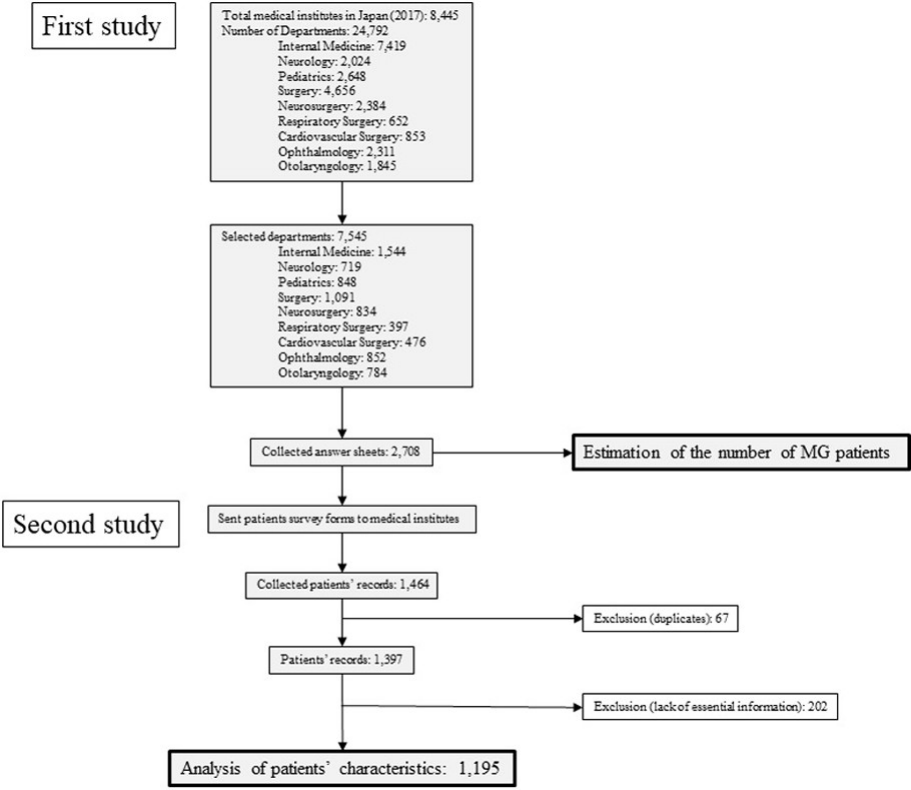
Flow chart of the epidemiological sturdy 2018. The study comprises the first survey and the second survey.

This study was supported (in part) by a Health and Labour Sciences Research Grant on Rare and Intractable Diseases (Taskforce of Validation of Evidence-based Diagnosis and Guidelines, and Impact on Quality of Life (QOL) in Patients with Neuroimmunological Diseases) from MHLW (Grant number: 20FC1030). The survey followed the Survey Manual of Study on Continuous Epidemiological Data Collection and Intractable Diseases from MHLW, third edition [19]. We summarized the flow of the study procedure in Fig 1.

### Protocol approvals, registrations, and patient consent

We located the study center at the Health Service Center of Kanazawa University (Kanazawa, Japan), and the Kanazawa University Medical Ethics Committee approved the study protocol (2017–292). However, the study was retrospective based on the medical records, and the correspondence tables of patients were stored in the medical departments that participated in this study. Therefore, we waived written informed consent from patients. Instead, we provided posters to the medical departments, which announced the ongoing study and gave patients a chance not to include their data in the survey.

### The first survey

We performed the first survey to estimate the prevalence of patients. MHLW provides diagnostic criteria of MG and severity classification on the Japan Intractable Diseases Information Center (https://www.nanbyou.or.jp/entry/272). We used these criteria for the survey (Table 1).

**Table 1:** Diagnostic criteria for MG.

1	Having the following subjective and objective symptoms, accompanied by easy fatigue and circadian fluctuations	
	1	Blepharoptosis
	2	Eye movement restriction
	3	Facial muscle weakness
	4	Dysarthria
	5	Dysphagia
	6	Masticatory disorders
	7	Neck muscle weakness
	8	Muscle weakness in extremities and trunk
	9	Dyspnea
2	Positive: one of the following autoantibodies	
	1	Anti-AChR antibody
	2	Anti-MuSK antibody
3	Physiological findings indicating neuromuscular junction disorder by the following tests	
	1	Low-frequency repetitive nerve stimulation evoked electromyogram
	2	Edrophonium test (Evaluate using objective indices such as eye movement disorders and low-frequency repetitive stimulation-induced electromyogram)
	3	Single fiber EMG (SFEMG)
4	Differential diagnosis	
		Differentiation candidates are all diseases that cause ophthalmoplegia, limb muscle weakness, and dysphagia / respiratory disorders.
		Lambert-Eaton myasthenia syndrome, Muscular dystrophy (Becker type, Limb-girdle type, Facio-scapulohumeral type), Polymyositis, Periodic paralysis, Hyperthyroidism, Mitochondrial encephalomyopathy, Progressive ophthalmoplegia, Guillain-Barre syndrome, Polyneuritis, Oculomotor nerve palsy, Tolosa-Hunt syndrome, Brainstem tumors and vascular disorders, Brainstem encephalitis, Herpes simplex and another viral encephalitis, Basal meningitis, Temporal arteritis, Wernicke’s encephalopathy, Lee’s encephalopathy, Diabetic extraocular palsy, Vasculitis, Neuro-Behcet’s disease, Sarcoidosis, Multiple sclerosis, acute disseminated encephalomyelitis, Fisher syndrome, Congenital myasthenia syndrome, Congenital myopathy, Myotony, Blepharospasm, Apraxia of the eyelid, Amyotrophic lateral sclerosis, Eyelid skin laxity, Botulism
Diagnosis criterion		
A	At least one of [1] and one of [2] are satisfied	
B	At least one of [1] and any of [3], plus exclude all diseases listed in [4]	

AChR: acetylcholine receptor, MuSK: muscle-specific tyrosine kinase

According to the MHLW, the total number of medical institutions in Japan in 2017 was 8,445 (https://www.mhlw.go.jp/toukei/saikin/hw/iryosd/17/). Following instructions of the survey manual, the numbers of medical departments to be investigated were as follows: Neurology; 2,024, Internal medicine; 7,419, Pediatrics; 2,648, Surgery; 4,656, Neurosurgery; 2,384, Respiratory Surgery; 652, Cardiovascular Surgery; 853, Ophthalmology; 2,311, and Otolaryngology; 1,845. From these departments, we selected 100% of university hospitals, 100% of hospitals having more than or equal to 500 beds, 80% of hospitals having 400–499 beds, 40% of hospitals having 300–399 beds, 20% of hospitals having 200–299 beds, 10% of hospitals having 100–199 beds and 5% of hospitals having less than or equal 99 beds. We also selected four hospitals that see many MG patients. One of the authors (YNa) selected the candidates randomly and prepared the list of departments. As a result, we selected the medical departments to send the first survey: Internal medicine;1544, Neurology; 719, Pediatrics; 848. Surgery; 1,091, Neurosurgery; 834, Respiratory surgery; 397, Cardiovascular surgery; 476, Ophthalmology; 852 and Otolaryngology; 784. The total number of departments to send the survey sheet on March 30, 2018, was 7,545. To these departments, we sent survey sheets asking the number of MG patients who visited their places from January 1, 2017, to December 31, 2017. We also asked the number of patients diagnosed with MG at each department from January 1, 2015, to December 31, 2017, to send the second survey sheet (S1 Table). We restricted the range of the second survey to three years to ensure the accuracy and reliability of the case records.

### The second survey

We sent the second survey form to the medical department that responded to the first survey. The documents include anonymous case records, which ask for clinical information of patients diagnosed as MG from January 1, 2015, to December 31, 2017. The correspondence tables of the secondary survey were stored in medical departments. Therefore, the data used were de-identified and anonymized before we had access. The second form included patient symptoms, autoantibodies titers, examinations, clinical severities, the method for thymectomy, thymic images and pathologies, therapies, and other information (S1 Table). Titers of AChRAb were measured by immunoprecipitation assay using a kit of Cosmic Corporation Co., Ltd. (Tokyo, Japan), which was an immunoprecipitation assay by ^125^I-labeled α-bungarotoxin and TE671 extract. TE671 is a subline of rhabdomyosarcoma cell line RD [20]. Titers of MuSKAb were measured by ^125^I-labeled immunoprecipitation assay kit provided by Cosmic Corporation Co., Ltd.

### Data analysis

We calculated the estimated number of patients by the formulae indicated in the Survey Manual, third edition [19]. In addition, we calculated the prevalence rate per 100,000 using the Japanese population in 2017 reported by the Statistics Bureau of Japan (n: 126,706,000, https://www.stat.go.jp/data/jinsui/2017np/index.html).

We used the Shapiro-Wilk test to evaluate the distribution of continuous data in this study and found it was non-normal. So we expressed the distribution of continuous data with median [interquartile range (IQR)]. To compare two continuous data, we used Wilcoxon-Mann-Whitney test (WMW). To compare multiple continuous data, we utilized Steel-Dwass All Pairwise Comparison. Finally, we used Fisher’s exact test to compare categorical data between MG categories, followed by multivariate logistic regression analyses. We utilized JMP 16.2.0 (SAS Institute, Japan, Tokyo, Japan) for the statistical analysis. All patients had information about age, sex, onset age, initial symptom, Myasthenia Gravis Foundation of America (MGFA) clinical classification, myasthenia gravis activities of daily living profile (MG-ADL), AChRAb, MuSKAb, and thymectomy. There were missing data in some information; however, we proceeded with the analyses of these records.

## Results

### First survey

#### Estimated number and prevalence of patients

We received 2,708 survey forms from medical departments (percentage of replies: 35.9%). As a result, the number of MG patients in Japan in 2017 was 29,210 (95%CI: 26,030–32,390). The prevalence of the 100,000 population was 23.1 (95% CI: 20.5–25.6). According to the previous nationwide study for 2006 [18], the estimated number was 15,100 (95% CI: 13,900–16,300), and the prevalence rate was 11.8 per 100,000 (95% CI: 10.9–12.7). As a result, the number of patients doubled in the last eleven years.

### Second survey

We obtained 1,464 clinical records from medical departments and carefully checked the sheets. We excluded 67 records because of duplication. We also excluded five records without information on gender and eleven without onset age. We excluded 202 patients who received a diagnosis of MG before 2015 or in 2018. The final number of patient records was 1,195 (Fig 1).

#### Onset age

**Fig 2:**
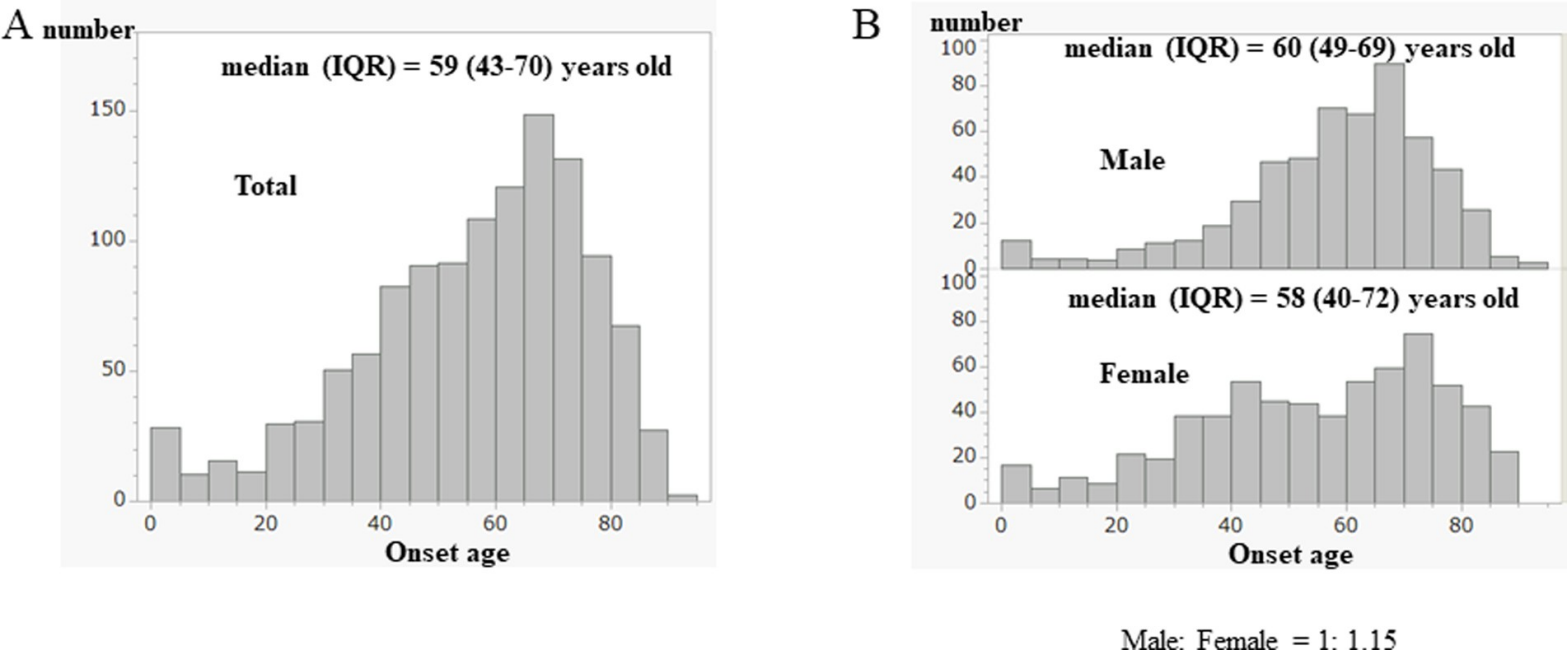
Distribution of onset age of total MG patients. (A) Histogram of onset age of total patients. Median (IQR) = 59 (43–70) years old. (B) Histogram of onset age by sex. Median (IQR): male; 60 (49–69) years old, female; 58 (40–72) years old. The male was significantly older than the female [Wilcoxon-Mann-Whitney test (WMW), p = 0.0299].

The median (IQR) of the onset age of total patients was 59 (43–70) years old (Fig 2A).

Notably, there was a small peak of fewer than five years old. The ratio of male: to female was 1: 1.15. The median (IQR) onset age for male patients was 60 (49–69) years old, and that of the female was 58 (40–72) years old (Fig 2B). The male patients were significantly older than the females (WMW, p = 0.0299). We compared our results with the previous data after the report of Murai et al. [18] (Table 2).

**Table 2:** Comparison of demographic features among nationwide surveys in Japan.

	1973	1987	2006	2018 (Current Study)
Estimated number of patients	ND	6,000	15,100	29,210
Prevalence rate (per 100,000)	1.35	5.1	11.8	23.1
Male: Female (ratio)	1:2.1	1:1.85	1:1.7	1:1.15
Infantile-onset (onset-age of 0–4 years) (%)	ND	10.1	7.0	2.3
Late- and elderly-onset (onset-age of 50 years or older) (%)	ND	28.8	41.7	66.1
Occurrence with families (%)	2.0	0.8	0.7	0.8
Ocular type (MGFA Clinical Classification Class I) (%)	ND	40.1	35.7	36.8
Concurrence of thymoma (%)	10.6	21.1	32.0	22.4
History of crisis (%)	16.0	14.8	13.3	6.9

ND: not determined, MGFA: Myasthenia Gravis Foundation of America

The prevalence rate increased steadily, and the predominance of females decreased over the years. Remarkable changes were decreasing percentile of infantile-onset (onset-age of 0–4 years) and the increasing percentile of late- and elderly-onset (onset-age of 50 years or older). Additionally, we observed the slightest history of the crisis in the current study.

#### Classification of patients by autoantibodies and existence of thymoma

Thymoma existed in 26.4% (265/1003) of AChRAb(+), 0% (0/34) of MuSKAb(+) and 1.3% (2/154) of DN. We excluded two patients with thymoma from DN and classified patients into four categories; 1) AChRAb(+) thymoma(Tm)(-), 2) AChRAb(+)Tm(+), 3) MuSKAb(+), 4) DN. In the antibody testing process, clinicians test MuSKAb in patients of AChRAb(-). However, 272 of 1007 AChRAb(+) (27.0%) were tested for MuSKAb and found 4/272 (1.5%) were positive for both AChRAb and MuSKAb. Three of four patients had low titers of MuSKAb [AChRAb: MuSKAb, 8.9:100, 4.0: 0.01, 52: 0.02, 0.3: 0.02 (nmol/L)]. Therefore, we excluded these four patients from further analysis. As a result, the total number of patients we examined further was 1189. Some patients have anonymous data in several questions; however, we proceed with these patient records.

#### Clinical characteristics of patients by categories

**Fig 3:**
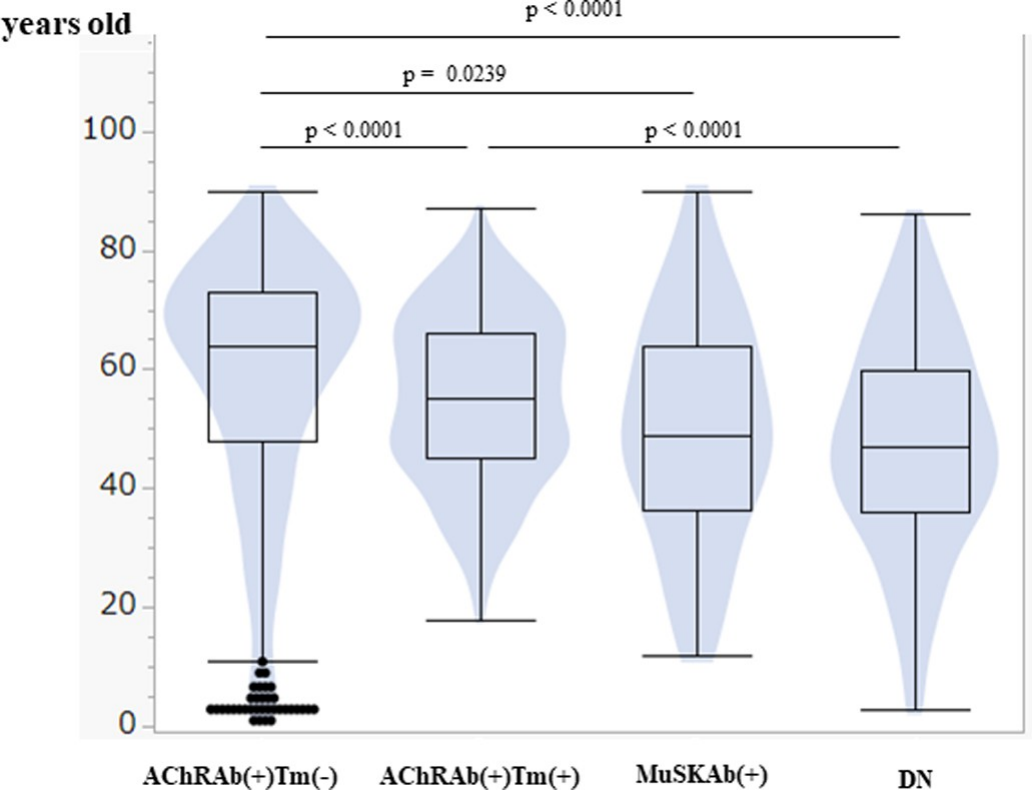
Onset age by categories. Box plot and violin plot of onset age by categories. The horizontal line within the box represents the median value. The box ends to define the 25th and 75th quantiles, expressed as the first and third quartiles. The lines that extend from the box end are whiskers. The whiskers extend from the ends of the box to the outermost data point that falls within these distances. 1st quartile–1.5 x (interquartile range), 3rd quartile+1.5 x (interquartile range). For statistical analysis, Steel-Dwass All Pairwise Comparison was performed.

The onset age [median (IQR)] of AChRAb(+)Tm(-) was 64 (48–73) years old, and AChRb(+)Tm(+) was 55 (45–66), MuSKAb(+) was 49 (36–64), DN was 47 (35–60) year old (Fig 3).

The onset ages of AChRAb(+) patients were higher than those of other categories. Interestingly, patients less than five years old were in AChRAb(+)Tm(-). In addition, the female was predominant in MuSKAb(+) (Table 3).

**Table 3:** Male to female ratio by categories.

	AChRAb(+)Tm (-)	AChRAb(+)Tm(+)	MuSKAb(+)	DN	Chi-square test	
	(n = 738)	(n = 265)	(n = 34)	(n = 152)	χ^2^	p
Gender no (%)						
Male	363 (49.2)	114 (43.0)	10 (29.4)	66 (43.4)	8.002	0.0460^a^
Female	375 (50.8)	151 (57.0)	24 (70.6)	86 (56.6)		

AChRAb(+)Tm(-): anti-acetylcholine receptor antibody (+) and thymoma (-), AChRAb(+)Tm(+): anti-acetylcholine receptor antibody (+) and thymoma (+), MuSKAb(+): anti-muscle-specific kinase antibody (+), DN: anti-acetylcholine receptor antibody (-) and anti-muscle-specific kinase antibody (-)

MGFA Clinical Classification Class I (ocular type) percentile was significantly low in MuSKAb(+) (Table 4).

**Table 4:** Initial symptoms of patients.

	AChRAb(+)Tm(-)	AChRAb(+)Tm(+)	MuSKAb(+)	DN	Chi-square test	
	(n = 738)	(n = 265)	(n = 34)	(n = 152)	χ^2^	p
Ocular type, n (%)	319 (43.2)	61 (23.0)	2 (5.9)	56 (36.8)	48.691	<0.0001
Initial symptoms n (%)						
Blepharoptosis	553 (74.9)	188 (70.9)	15 (44.1)	103 (67.8)	17.820	0.0005
Ophthalmoplegia	350 (47.4)	94 (35.5)	12 (35.3)	64 (42.1)	12.587	0.0056
Facial weakness	25 (3.4)	15 (5.7)	2 (5.9)	8 (5.3)	3.278	0.3508
Dysarthria	83 (13.3)	32 (12.1)	7 (20.6)	15 (9.9)	3.284	0.3499
Dysphagia	89 (12.1)	43 (16.2)	12 (35.3)	12 (7.9)	21.278	<0.0001
Chewing weakness	25 (3.4)	17 (6.4)	3 (8.9)	5 (3.3)	6.554	0.0876
Neck weakness	42 (5.8)	28 (10.6)	6 (17.6)	14 (9.2)	11.992	0.0074
Limbs and trunk weakness	100 (13.6)	57 (21.5)	10 (29.4)	21 (20.4)	16.143	0.0017
Dyspnea	19 (2.6)	10 (3.8)	3 (8.8)	4 (2.6)		

AChRAb(+)Tm(-): anti-acetylcholine receptor antibody (+) and thymoma (-), AChRAb(+)Tm(+): anti-acetylcholine receptor antibody (+) and thymoma (+), MuSKAb(+): anti-muscle-specific kinase antibody (+), DN: anti-acetylcholine receptor antibody (-) and anti-muscle-specific kinase antibody (-)

MuSKAb(+) had lesser ocular symptoms and more dysphagia than other categories.

**Fig 4:**
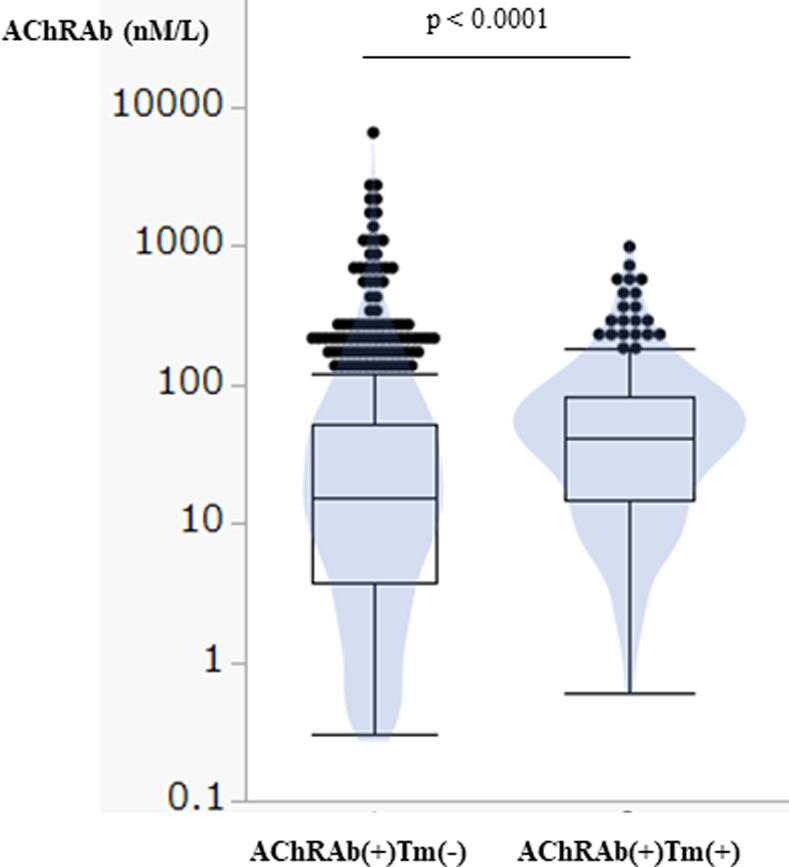
Maximum AChRAb titer of AChRb(+)Tm(-) and AChRAb(+)Tm(+). Box and violin plots of maximum titer of AChRAb of anti-acetylcholine receptor antibody (AChRb) (+) thymoma (Tm) (-) and AChRAb(+)Tm(+). The horizontal line within the box represents the median value. The box ends to define the 25th and 75th quantiles, also expressed as the first and third quartiles, respectively. The lines that extend from the box end are whiskers. The whiskers extend from the ends of the box to the outermost data point that falls within these distances. 1st quartile–1.5 x (interquartile range), 3rd quartile+1.5 x (interquartile range). For statistical analysis, we used Wilcoxon-Mann-Whitney test (WMW).

The titers of maximum AChRAb were significantly higher in patients with thymoma [median (IQR); 41 (14.6–80.1)] than without thymoma [median (IQR); 15 (3.7–51)] (WMW: p<0.0001) (Fig 4).

We observed the lesser positive result of the repetitive nerve stimulation test (RNST) and higher positive results for single fiber electromyogram (SFEMG) in DN (Table 5). Additionally, the positive percentile of the edrophonium test was relatively low in MuSKAb(+).

**Table 5:** Examination to evaluate NMJ function.

	AChRAb(+)Tm(-)	AChRAb(+)Tm(+)	MuSKAb (+)	DN	Chi-square test		
	(n = 738)	(n = 265)	(n = 34)	(n = 152)	χ^2^		p
Examination, n/n-total (%*)							
RNST	409/592 (69.1)	162/203 (79.8)	21/30 (70.0)	60/140 (42.9)	75.110	<0.0001	
SFEMG	55/101 (54.5)	15/30 (50.0)	0/3 (0.0)	40/53 (75.5)	66.680	<0.0001	
Edrophonium test	488/513 (95.1)	165/173(95.4)	22/29 (75.9)	118/139 (84.9)	76.808	<0.0001	

*% refers to the proportion of patients who tested positive for each assessment.

AChRAb(+)Tm(-): anti-acetylcholine receptor antibody (+) and thymoma (-), AChRAb(+)Tm(+): anti-acetylcholine receptor antibody (+) and thymoma (+), MuSKAb(+): anti-muscle-specific kinase antibody (+), DN: anti-acetylcholine receptor antibody (-) and anti-muscle-specific kinase antibody (-)

RNST: repetitive nerve stimulation test, SFEMG: single-fiber electromyography.

The incidence of Graves’ disease was relatively high in DN (Table 6). On the other hand, MuSKAb (+) did not accompany other autoimmune diseases. In addition, the incidence of family history was low in all categories.

**Table 6:** Comorbidities and family history of MG.

	AChRAb(+)Tm (-)	AChRAb(+)Tm(+)	MuSKAb(+)	DN	Chi-square test	
	(n = 738)	(n = 265)	(n = 34)	(n = 152)	χ^2^	p
Comorbidities n (%)						
Rheumatoid arthritis	13 (1.8)	2 (0.8)	0 (0.0)	2 (1.3)		
Hashimoto’s disease	37 (5.0)	7 (2.6)	0 (0.0)	8 (5.3)	4.854	0.1828
Graves’ disease	29 (3.9)	4 (1.5)	0 (0.0)	13 (8.6)	14.818	0.0025
SLE	5 (0.7)	0 (0.0)	0 (0.0)	0 (0.0)		
Pure red cell aplasia	0 (0.0)	0 (0.0)	0 (0.0)	0 (0.0)		
Multiple sclerosis	0 (0.0)	0 (0.0)	0 (0.0)	1 (0.7)		
Family history of MG n (%)	7 (0.9)	0 (0.0)	0 (0.0)	2 (1.3)		

AChRAb(+)Tm(-): anti-acetylcholine receptor antibody (+) and thymoma (-), AChRAb(+)Tm(+): anti-acetylcholine receptor antibody (+) and thymoma (+), MuSKAb(+): anti-muscle-specific kinase antibody (+), DN: anti-acetylcholine receptor antibody (-) and anti-muscle-specific kinase antibody (-)

SLE: systemic lupus erythematosus

We compared the characteristics of each category by Fisher’s exact test followed by multivariate logistic regression analysis using the data of sex, initial symptoms, and examinations (RNST and Edrophonium test). We omitted SFEMG from examinations because the number of tests was limited. In comparison between AChRAb(+)Tm(-) and AChRAb(+)Tm(+), female was significantly dominant in AChRAb(+)Tm(+) (OR; 1.4969, p = 0.0469). Ophthalmoplegia was significantly often in AChRAb(+)Tm(-) (OR; 1.7610, p = 0.0064). Chewing weakness was significantly less in AChRAb(+)Tm(-) (OR; 0.2746, p = 0.0212). The positive RNST was significantly lower in AChRAb(+)Tm(-) (OR; 0.6054, p = 0.0412). MuSKAb(+) had lower ophthalmoplegia, higher dysphagia, and lower positive Edrophonium test. DN had significantly lower RNST positive compared to other categories.

To further analyze the evidence of NMJ impairment in DN, we studied the results of RNST, SFEMG, and Edrophonium test of DN. As a result, the outcomes of the three tests were inconsistent, indicating the complexities of DN’s pathophysiologies.

**Fig 5:**
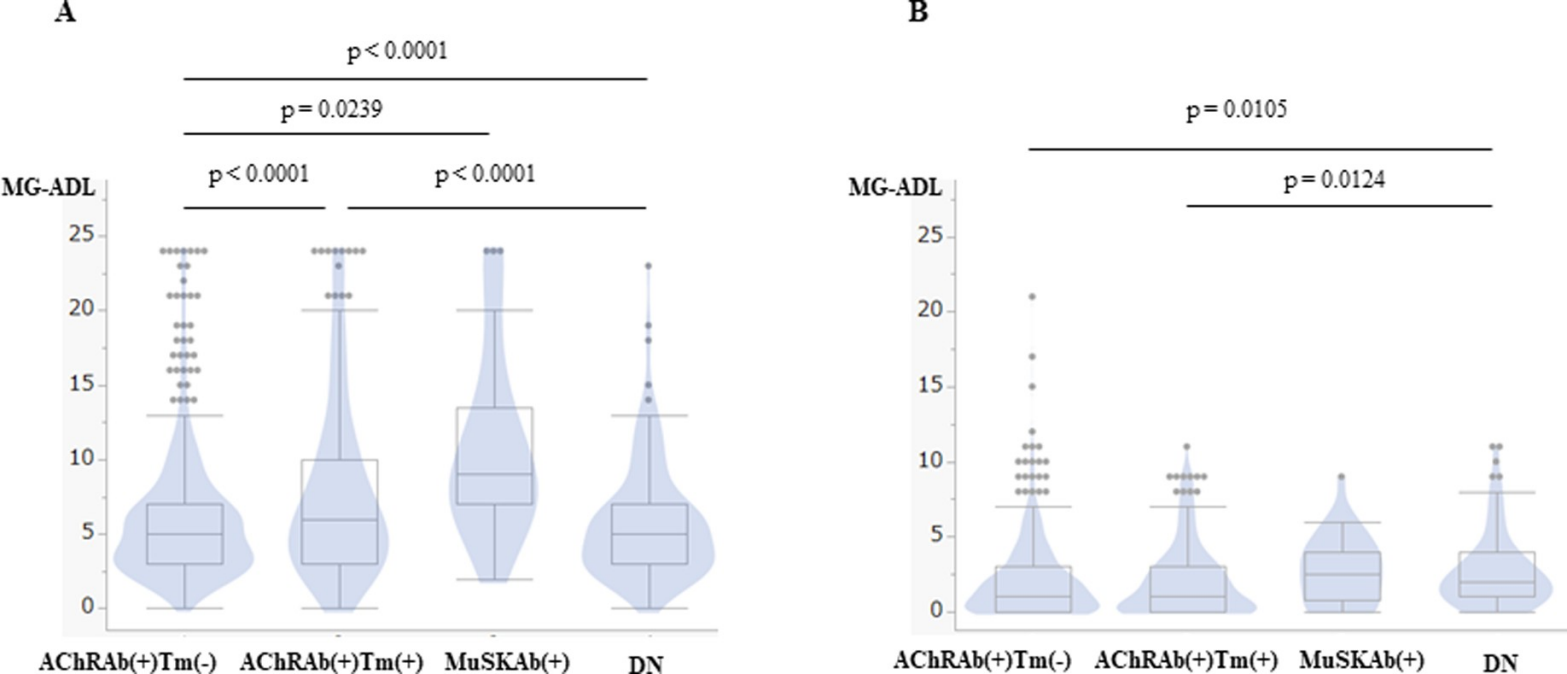
Myasthenia gravis activities of daily living profile by categories. **(A)** Box and violin plots of myasthenia gravis activities of daily living profile (MG-ADL) at the severest state. (B) Box and violin plots of MG-ADL at the current state. The horizontal line within the box represents the median value. The box ends represent the 25th and 75th quantiles, expressed as the first and third quartiles. The lines that extend from the box end are whiskers. The whiskers extend from the ends of the box to the outermost data point that falls within these distances. 1st quartile–1.5 x (interquartile range), 3rd quartile+1.5 x (interquartile range). For statistical analysis, Steel-Dwass All Pairwise Comparison was performed.

In addition, MuSKAb(+) had a higher MG-ADL than others in the severest states (Fig 5A). However, the current MG-ADL was low in all categories (Fig 5B).

**Fig 6:**
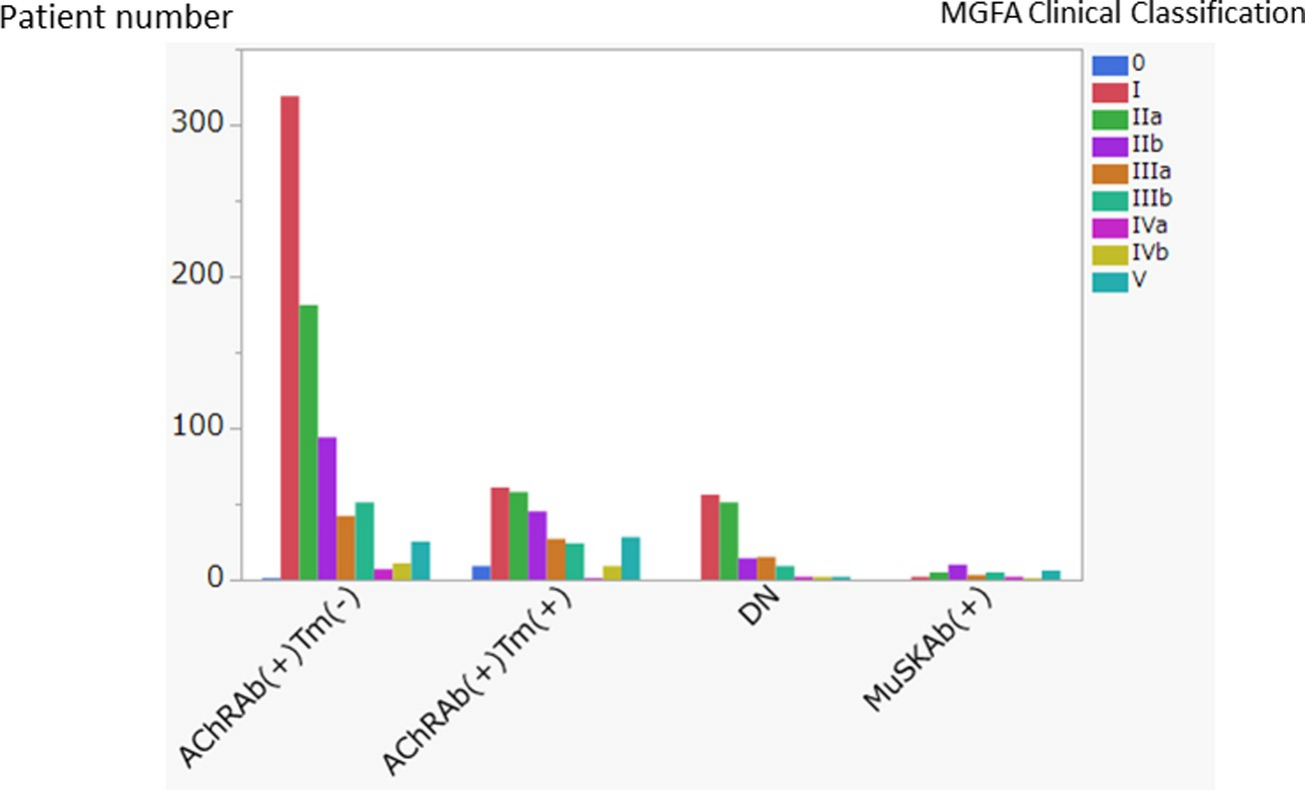
Myasthenia Gravis Foundation of America (MGFA) clinical classification by myasthenia gravis categories. Bar graph plot of Myasthenia Gravis Foundation of America (MGFA) clinical classification of myasthenia gravis (MG) categories. The clinical classification was done according to maximum disease severity. The X-axis indicates categories of MG. Y-axis indicates the patients’ number.

The graphical plot of MGFA clinical classification indicates further insight into each category’s clinical features (Fig 6).

The frequent type in AChRAb(+)Tm(-), AChRAbTm(+), and DN was MGFA class I (ocular type), followed by IIa, then IIb. The frequent type of MuSKAb(+) was IIb. MGFA class 0 indicates without symptom.

Therapy and outcome 99.2% of AChRAb(+)Tm(+) patients received thymectomy. On the other hand, no patients in MuSKAb (+) and a few of DN received thymectomy (Table 7).

**Table 7:** Therapies and outcome by categories.

	AChRAb(+)Tm(-)	AChRAb(+)Tm(+)	MuSKAb(+)	DN	Chi-square test	
	(n = 738)	(n = 265)	(n = 34)	(n = 152)	χ^2^	p
Thymectomy: n (%)	102 (15.4)	263 (99.2)	0 (0.0)	8 (5.7)	104.0	<0.0001
Medication: n (%)						
AChEI	592 (80.2)	196 (74.0)	14 (41.2)	127 (83.6)	34.366	<0.0001
PSL	493 (66.8)	196 (74.0)	32 (94.1)	98 (64.5)	16.160	0.0011
Tacrolimus	301 (40.8)	142 (53.6)	26 (76.5)	49 (32.2)	36.047	<0.0001
Ciclosporin	30 (4.1)	12 (4.5)	3 (8.8)	5 (3.3)	2.221	0.5278
Steroid pulse n (%)	145 (19.8)	59 (22.7)	14 (41.2)	34 (22.4)	9.234	0.0263
Plasmapheresis: n (%)	66 (9.1)	56 (21.1)	11 (33.3)	23 (15.2)	37.605	<0.0001
IVIg: n (%)	128 (17.5)	82 (30.9)	17 (50.0)	35 (23.0)	36.351	<0.0001
Crisis: n (%)						
Post-operation	7 (1.0)	18 (7.1)	NA	0 (0.0)	35.318	<0.0001
Post-infection	6 (0.8)	8 (3.2)	0 (0.0)	2 (1.3)	7.630	0.0543
Others	20 (2.8)	13 (5.1)	4 (13.3)	1 (0.7)	14.301	0.0025
Exacerbation: n (%)	87 (11.9)	46 (17.4)	5 (14.7)	16 (10.5)	6.589	0.0862
Death: n (%)						
MG	2 (0.3)	0 (0.0)	1 (2.9)	0 (0.0)		
other reasons	6 (0.8)	5 (1.9)	1 (2.9)	1 (0.7)		

AChRAb(+)Tm(-): anti-acetylcholine receptor antibody (+) and thymoma (-), AChRAb(+)Tm(+): anti-acetylcholine receptor antibody (+) and thymoma (+), MuSKAb(+): anti-muscle-specific kinase antibody (+), DN: anti-acetylcholine receptor antibody (-) and anti-muscle-specific kinase antibody (-), AChEI: acetylcholine esterase inhibitor, PSL: prednisolone, IVIg: intravenous immunoglobulin

Regarding surgical methods, video-assisted thymectomy was a primary method in AChRAb(+)Tm(-); however, AChRAb(+)Tm(+) received extended thymectomy frequently (S2 Table). Masaoka Staging and WHO Histologic Classification of thymoma are in S3 and S4 Tables.

**Fig 7:**
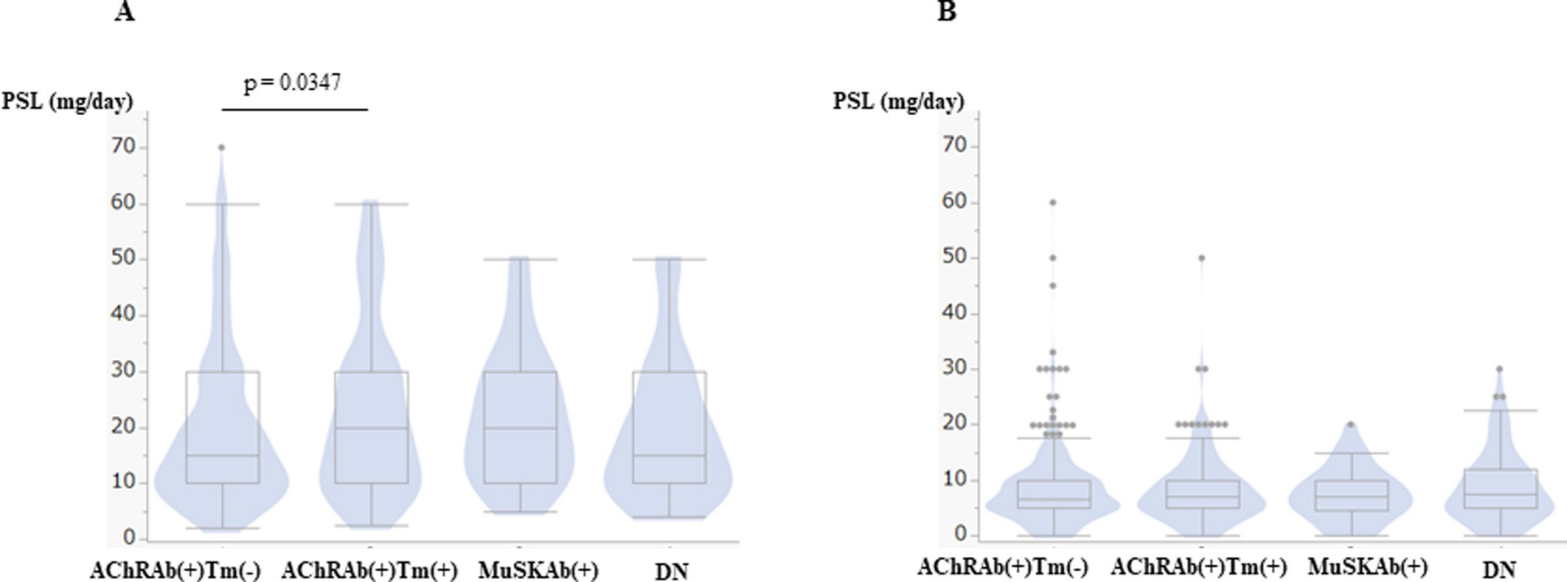
Prednisolone (PSL) dose at the maximum and the current states. (A) Box and violin plot plots of prednisolone (PSL) at maximum dose. (B) Box and violin plot of PSL at the current dose. The horizontal line within the box represents the median value. The box ends represent the 25th and 75th quantiles, expressed as the first and third quartiles. The lines that extend from the box end are whiskers. The whiskers extend from the ends of the box to the outermost data point that falls within these distances. 1st quartile–1.5 x (interquartile range), 3rd quartile+1.5 x (interquartile range). For statistical analysis, Steel-Dwass All Pairwise Comparison was performed.

Acetylcholine esterase inhibitor (AChEI) is frequently subscribed (Table 7); however, MuSKAb(+) received significantly less AChEI (p<0.0001). On the other hand, the maximum prednisolone (PSL) prescription was relatively higher in AChRAb(+)Tm(+), and MuSKAb(+) (Fig 7A).

AChRAb(+)Tm(+) received a significantly higher dose of PSL than AChRAb(+)Tm(-) (Steel-Dwass All Pairwise Comparison, p = 0.0347). However, there was no difference in the current dose of PSL in categories, and the median doses were less than 10 mg/day (Fig 7B). Additionally, the prescription of tacrolimus was more frequent in MuSKAb(+), and the usage of steroid pulse, plasmapheresis, and intravenous immunoglobulin (IVIg) were also frequent in MuSKAb(+).

The percentile of a crisis of post-operation was recognizable in AChRAb (+)Tm(+). The mortality by MG was not remarkable.

## Discussion

MG is a well-recognized autoimmune disease in neurological disorders. However, repeated epidemiological surveys with the same procedure in one region are not frequently performed. Ideally, an epidemiological survey should be performed repeatedly for a particular duration to examine prevalence and clinical features changes. Therefore, we conducted a nationwide study to elucidate MG prevalence and clinical features in Japan after 12 years of a previous one by Murai et al. [18] and found a doubled prevalence, even male: female ratio and elderly onset ages. A systematic review by Carr et al. examined 55 studies performed using reports from 1950 to 2007, and they estimated a pooled prevalence rate: 77.7 per million persons (CI: 64.0–94.3) [21]. McGrogan et al. reported a systemic review of the literature published between 1980 and 2007 and found the incidence rates between 3.0 and 30.0/1,000,000/year [22]. They also claimed that incidence rates have increased over time owing to a greater awareness of the disease and improved methods of diagnosis. Otherwise, Cetin et al. reported MG prevalence in Austria in 2009 as 15.69 per 100,000 (95% CI 13.16–19.42) and claimed rising prevalence in an aging society [23]. Casetta et al. reported the changing incidence pattern with an increase in the frequency of late-onset and a decrease of early-onset MG, which was attributable to unknown environmental factors [24]. Park et al. reported increasing patient numbers in South Korea. They examined the National Health Insurance database in South Korea and found that the incidence rate in 2010 was 1.18/100,000 and that in 2018 was 1.81/100,000 [25]. Lerner et al. reported that the world incidence and prevalence of autoimmune diseases increased [26] and presented the possible cause of intestinal tight junction permeability changes associated with industrial food additives [27]. Our study showed a similar tendency in the Japanese population. It is also notable that an incidence peak that existed in equal to or less than the five-year-old population in Japan was not evident compared to the previous study [18]. Matsuki et al. studied human leukocyte antigen (HLA) antigens in 104 patients and 41 families with MG and reported the increased DR9 and Drw13 in the patients who developed MG before three years old [28]. In their study, they excluded patients with thymoma. On the other hand, Suzuki et al. studied MG susceptibility in Japanese and concluded that immunogenetic backgrounds in Japanese adult MG patients were heterogeneous and different from Caucasian patients [29]. Interestingly, Saruhan-Direskeneli reported genetic heterogeneity with the HLA region in early-onset and late-onset AChRAb(+) and MuSKAb (+) at the genome-wide level in Turkey [30]. Maniaol, A.H. et al. reported that late-onset MG (LOMG) (onset≥60 years) is associated with HLA DRB1*15:01 in the Norwegian population [31]. Spagni G. et al. reported that HLA DRB1*07-DQB1*02 haplotype as a predisposing factor tor the development of generalized AChRAb(+)Tm(-) LOMG in the Italian population [32]. Further, genome-wide association study confirmed TNFRSF11A and identified ZBTB10 and three HLA association with AChRAb(+) LOMG (onset≥50 years old) [33]. Therefore, it is suspectable that AChRAb(+)Tm(-) LOMG has genetic background in its onset. Pakzad, Z. et al. reported an increasing incidence of MG in Canada in the population over 65 years old without alteration in less than 64 years old from 1986 to 2006 [14]. Our observation in Japan showed that MG patients of late- and elderly-onset (onset ≥50 years old) was doubled from 2006 to 2018. Accordingly, the prevalence of MG especially in the subtype of AChRAb(+)Tm(-)LOMG has been steadely increasing. There might be environmental factors as well as genetic factors that we have to closely study.

On the other hand, Belimezi et al. studied age at sampling and sex distribution of AChRAb(+) and MuSKAb(+) in a Greek population and found a peak of around 10-year-old in both AChRAb and MuSKAb MGs [34]. There might be unknown factors related to the onset of juvenile MG. However, our study showed no peak in MuSKAb(+) at around ten years old. The genetic factors might differ in ethnicities or regions, which will be one of the study targets.

The clinical features classified by autoantibody profile and the existence of thymoma are not been thoroughly studied in one nationwide survey before. Onset age by the classification brought us several essential information. Firstly, AChRAb (+) Tm (-) had older onset age than other categories. Secondly, fewer than five years old peaked existed only in the AChRAb(+)Tm(-). These findings suggest that AChRAb(+)Tm(-) has unique pathogenesis from AChRAb(+)Tm(+).

Interestingly, thymoma MG has unique immunological characteristics, including serum interleukin (IL)-12p40 elevation and anti-IL-12p40 antibody [35], and anti-interferon-α (IFNα) antibody [36]. Thymoma is also associated with other autoimmune diseases [13]. From our study, the MG-ADL at the severest state is different with thymoma and without thymoma, and the titer of AChRAb is significantly higher in thymoma than in non-thymoma. These findings recommend thinking of these two types as different categories. However, the current MG-ADL was not different in all categories. As a result, the prognosis of AChRAb(+)Tm(+) is favorable as AChRAb(+)Tm(-) [37].

The MuSKAb(+) has clinical characteristics different from the AChRAb(+). The onset age is younger than the AChRAb(+), and females are predominant, as previously reported [34, 38]. MGFA clinical classification class I (ocular type) is less than ten percent in MuSKAb(+). The MG-ADL of MuSKAb(+) at the severest state was higher than other categories. We also find the bulbar symptom predominancy in MuSKAb(+) as in the previous [38, 39]. No patients of MuSKAb(+) received thymectomy in our survey. Remarkably, MuSKAb(+) has no other complications of autoimmune diseases. Moreover, the MuSKAb(+) received less frequent AChEI. Cholinergic hyperactivity is common in MuSKAb(+) [40]. Therefore neurologists avoided prescriptions of AChEI that might induce a cholinergic crisis. In addition, MuSKAb received a significantly higher frequency of PSL, tacrolimus, plasma exchange and IVIg than other categories. Tacrolimus is an immunosuppressant that inhibits T-cell activation via disruption of calcineurin signaling [16]. It is prescribed for MG patients in Japan to enhance the immunosuppressive effect of steroids and reduce their dose [16, 17]. In our study, MuSKAb(+) prognosis is favorable and is not different from other categories, different from the report of Deymeer et al. [39]. Zhao et al. reported a favorable outcome of MuSKAb(+) in Northwest China [41]. Comparing ethnicities, countries, and therapeutic usage of immunosuppressants is necessary for future studies.

The DN has a younger onset age than other categories. Initial symptoms did not distinguish DN from AChRAb(+). However, the examination results are variable. Therefore, DN must have consisted of several entities. Therefore, to suspect an autoimmune mechanism and introduce immunotherapy, it is necessary to prove the impairment of neuromuscular transmission failure by RNST and SFEMG

The therapeutic outcomes of all categories were favorable, and MG-ADL satisfied patient-acceptable symptom states (PASS) [42]. These are attributable to the availability of early serological testing and thymic imaging. AChRAb(+)Tm(+) predominantly received thymectomy. However, the thymectomy percentage of AChRAb(+)Tm(-) did not reach twenty percent. The Thymectomy Trial in Non-Thymomatous Myasthenia Gravis Patients Receiving Prednisone Therapy (MGTX) revealed that thymectomy effectively improved MG symptoms and reduced PSL doses [43–45]. Following the results, the American Academy of Neurology (AAN) recommends discussing thymectomy treatment with patients with AChRAb (+) generalized MG (Level B) [46]. Thus, physicians should note that the indication of thymectomy is a treatment option for patients of AChRAb (+) Tm (-). The AAN also recommends minimally invasive thymectomy techniques (Level B) [46]. In this study, the video-assisted method was the first choice in AChRAb (+)Tm(-), as Solis-Pazmino [47] recommended.

This study’s limitations are 1) inherent limitations of the epidemiological survey, 2) the relatively low recovery rate of the survey, and 3) no systematic comparison with other countries or ethnicities. The response rate to the first study of 35.9% was considered relatively low. However, we followed the Survey Manual of Study on Continuous Epidemiological Data Collection and Intractable Diseases from MHLW [19], which was designed to prevent biases in the nationwide epidemiological study. In addition, we used the qualified diagnostic criteria of MG by Taskforce of Validation of Evidence-based Diagnosis and Guidelines and Impact on Quality of Life (QOL) in Patients with Neuroimmunological Diseases, which the Japanese Society of Neurology endorsed. Therefore, the survey procedure supports the estimation of reliable prevalence in this study. The methodology of an epidemiological study is different among studies, which is a significant problem in comparison of specificities depending on ethnicities or countries. Nevertheless, we will keep track of the same methodology and perform repeated surveys, which may provide us with chronologically changing pictures of MG. Those data will contribute to studying the etiology and patients’ welfare in other countries.

Finally, a further epidemiological study with attention to MG categories combined with genetic analysis and the therapeutic status will contribute to identifying the etiology and environmental factors that affect the occurrence of MG.

## Supporting information

S1 Table Patient records for the second survey. (DOCX)

S2 Table Surgical method of thymectomy. (DOCX)

S3 Table Masaoka staging of thymoma. (DOCX)

S4 Table WHO histologic classification of thymoma. (DOCX)

S1 File Minimal data for the first survey. (XLSX)

S2 File Minimal data for the second survey. (XLSX)
