# Supplementary material for: Two-step nationwide epidemiological survey of myasthenia gravis in Japan 2018
Source: PLoS One. 2022 Sep 21;17(9):e0274161. doi: 10.1371/journal.pone.0274161 (PMC9491589; doi:10.1371/journal.pone.0274161)
Supplement: S2 Table — (DOCX) [file pone.0274161.s002.docx]

S2 Table. Surgical method of thymectomy

|  | **AChRAb(+)Tm (-)**  **(n = 738)** | **AChRAb(+)Tm (+)**  **(n = 265)** | **MuSKAb (+)**  **(n = 34)** | **DN**  **(n = 152)** |
| --- | --- | --- | --- | --- |
| n (%) |  |  |  |  |
| Simple | 1 (0.1) | 10 (3.8) | NA | 0 (0.0) |
| Extended | 43 (5.8) | 186 (70.2) | NA | 1 (0.7) |
| Video-assisted | 54 (7.3) | 59 (22.3) | NA | 7 (4.6) |
| Others | 2 (0.3) | 3 (1.1) | NA | 0 (0.0) |
| unknown | 638 (86.5) | 7 (2.6) | NA | 144 (94.7) |

AChRAb(+)Tm(-): anti-acetylcholine receptor antibody (+) and thymoma (-), AChRAb(+)Tm(+): anti-acetylcholine receptor antibody (+) and thymoma (+), MuSKAb(+): anti-muscle-specific kinase antibody (+), DN: anti-acetylcholine receptor antibody (-) and anti-muscle-specific kinase antibody (-), NA: Not applicable
