# Supplementary material for: Two-step nationwide epidemiological survey of myasthenia gravis in Japan 2018
Source: PLoS One. 2022 Sep 21;17(9):e0274161. doi: 10.1371/journal.pone.0274161 (PMC9491589; doi:10.1371/journal.pone.0274161)
Supplement: S3 Table — (DOCX) [file pone.0274161.s003.docx]

S3 Table. Masaoka staging of thymoma

|  | **n (%)**  **(total = 243)** |
| --- | --- |
| I | 86 (35.4) |
| II | 115 (47.3) |
| III | 26 (10.7) |
| IVa | 14 (5.8) |
| IVb | 2/243 (0.8) |
