# Supplementary material for: Two-step nationwide epidemiological survey of myasthenia gravis in Japan 2018
Source: PLoS One. 2022 Sep 21;17(9):e0274161. doi: 10.1371/journal.pone.0274161 (PMC9491589; doi:10.1371/journal.pone.0274161)
Supplement: S4 Table — (DOCX) [file pone.0274161.s004.docx]

S4 Table. WHO histologic classification of thymoma

| **Type** | **n (%)**  **(total = 243)** |
| --- | --- |
| A | 17 (7.0) |
| AB | 54 (22.1) |
| B1 | 49 (20.1) |
| B2 | 93 (38.1) |
| B3 | 31 (12.7) |
